# Discrete Element Simulation of the Relationship between Composition, ITZ Property, and Tensile Behavior of Eco-Friendly UHPC Matrix

**DOI:** 10.3390/ma16103844

**Published:** 2023-05-19

**Authors:** Xiang Zhou, Ye Shi, Qingchun Hu, Shen Zhang, Xihong Zhang, Lingzhen Meng

**Affiliations:** 1 School of Civil Engineering, Central South University, Changsha 410075, China; xiangzhou@csu.edu.cn; 2 Tianjin Key Laboratory of Civil Structure Protection and Reinforcing, Tianjin Chengjian University, Tianjin 300192, China; 3 School of Civil and Mechanical Engineering, Curtin University, Perth 2605, Australia; 4 School of Resources and Safety Engineering, Central South University, Changsha 410083, China

**Keywords:** mesoscopic mechanical model, eco-friendly UHPC matrix, discrete element method, ITZ, tensile performance

## Abstract

To combat global warming, the development of eco-friendly ultra-high performance concrete (UHPC) has become one of the current research hotspots. Understanding the relationship between composition and performance of eco-friendly UHPC from a meso-mechanical point will be of great significance in proposing a more scientific and effective mix design theory. In this paper, the 3D discrete element model (DEM) of an eco-friendly UHPC matrix was constructed. The mechanism of the effect of the interface transition zone (ITZ) properties on the tensile behavior of an eco-friendly UHPC matrix was studied. The relationship between composition, ITZ property, and tensile behavior of eco-friendly UHPC matrix was analyzed. The results show that ITZ strength influences the tensile strength and cracking behavior of eco-friendly UHPC matrix. The effect of ITZ on the tensile properties of eco-friendly UHPC matrix is more significant than that of normal concrete. The tensile strength of UHPC will be increased by 48% when the ITZ property is changed from normal condition to perfect. Improving the reactivity of the binder system of UHPC will improve the performance of ITZ. The cement content in UHPC was reduced from 80% to 35%, and the σ_ITZ_/σ_Paste_ was reduced from 0.7 to 0.32. Both nanomaterials and chemical activators can promote the hydration reaction of the binder material, which in turn leads to better ITZ strength and tensile properties for an eco-friendly UHPC matrix.

## 1. Introduction

Ultra-high performance concrete (UHPC) is a new type of cementitious material, which was developed by Larrard and Sedran in the 1990s [1]. UHPC has extremely high strength, good toughness, and excellent durability. Therefore, UHPC can meet the requirements of light weight, large span, and high durability of civil engineering structures. UHPC shows its unique superiority in high-rise buildings, large-span bridges, offshore platforms, floating structures, and other projects [2,3,4,5]. The amount of cement used in UHPC is generally 800–1200 kg/m^3^. Under the conditions of an ultra-low water-to-binder ratio, a large amount of cement is unhydrated, and the cement utilization rate is low, which will make its economic and environmental costs higher and limit its wider application. In order to solve this problem, eco-friendly UHPC has become one of the hot spots of current research. Currently, some results have been achieved in preparing eco-friendly UHPC by optimizing the binder material system [6,7,8]. However, from the perspective of micro-mechanics, the mechanism influencing the performance of eco-friendly UHPC needs to be further studied, especially the effect of ITZ properties. This is of great significance to the composition optimization and performance improvement of the eco-friendly UHPC.

Concrete is a multi-phase composite material containing aggregate, hydrated cement paste, and the interface transition zone (ITZ). From the perspective of micro-mechanics, the ITZ is considered to be the “weak link” in concrete and is an important factor affecting the performance of concrete. The interfaces in UHPC are mainly divided into two types: the interface between fiber and UHPC matrix and the interface between cement paste and aggregate. The mechanical properties of the interfacial bond between fiber and matrix will determine the strengthening and toughening effect of UHPC, while the interfacial properties of cement paste and aggregate will directly change the mechanical response and damage behavior of the UHPC matrix. The interface property between fiber and matrix can be directly studied by fiber pull-out test [9], push-out test [10], single fiber fragmentation test [11], and microbond test [12]. Among them, the pull-out test has the advantages of simplicity, economy, and easy operation. More importantly, the stress state of the fibers and matrix in the pull-out test is similar to the stress conditions of the bridging fibers and the cracked matrix during the cracking process of composite material. On this basis, many researchers have carried out a large number of experimental and theoretical studies on the influence of fiber types [13], shape characteristics [14], surface roughness [15], and other factors on its interface and achieved certain results [16,17]. Compared with the interface between fiber and matrix, the properties of the ITZ between cement paste and aggregate in the matrix are difficult to be obtained directly by laboratory test methods. Some studies have investigated the structural and micromechanical characteristics of the ITZ using modern analytical and measurement methods such as CT scanning and nanoindentation [18,19], and the relationship between the microscopic characteristics of ITZ and the macroscopic properties of concrete have been analyzed [20,21]. However, due to the limitations of experimental equipment and specimen preparation methods, the research using experimental methods is expensive, and the results are more discrete. On the other hand, the initial cracking strength of UHPC has a significant impact on material and structural cracking, which in turn has a critical impact on the long-term service performance and life cycle of the building structure. The initial cracking strength of UHPC is determined by the tensile properties of the UHPC matrix, which will be significantly influenced by the properties of ITZ between cement paste and aggregate [8]. Therefore, it is of practical significance to analyze the tensile mechanical behavior and damage mechanism of the UHPC matrix from the perspective of the ITZ.

The eco-friendly UHPC reduces the amount of cement by optimizing the binder material system. The complex binder system for eco-friendly UHPC results in significant changes in its hydration as well as in the formation and development of its microstructure. Therefore, the composition of the binder material system has a significant influence on the structure formation and microscopic mechanical properties of ITZ [22]. Although advanced techniques such as nanoindentation and scanning electron microscopy (SEM) can obtain the mechanical properties of ITZ at the micro-nano scale, the influence mechanism of ITZ properties on the mechanical characteristics and damage behavior of eco-friendly UHPC has not been clarified and needs further study. For this research gap, numerical simulation can provide an effective means to study the quantitative relationship between the macroscopic mechanical behavior of concrete and ITZ structure by quantifying the meso-mechanical properties of ITZ, and it provides a basis for studying the mechanism of the influence of composition on ITZ performance and seeking better means of ITZ modification. As a meshless particle-based simulation method, the discrete element method (DEM) has proven to be a reasonable and efficient method to simulate concrete due to its advantage of visually expressing the nonlinear damage process of the material when the contact fracture between elements. In the DEM model, concrete is considered a three-phase composite consisting of cement, aggregate, and the interfacial transition zone between them [23]. Among them, there are two main methods to characterize the ITZ: (Ⅰ) In order to reduce the large amount of computer work associated with particle number; the ITZ is simulated as the contact between aggregate and cement matrix particles without physical thickness and the mechanical properties of the ITZ are described as the weakened meso-mechanical parameters of the cement matrix. (Ⅱ) ITZ is defined as the collection of fine particles formed in a specific width around the aggregate, and this region has the same meso-mechanical properties as the mortar and the higher porosity. These two methods have been gradually accepted by many scholars and used in discrete element simulation of concrete [24,25,26]. Currently, DEM models of concrete constructed using these two methods mainly focus on investigating the effects of concrete mesostructure (aggregate gradation, aggregate shape, aggregate/mortar volume, ITZ strength, and macroscopic porosity) on the macroscopic mechanical properties of concrete [27,28,29,30,31]. Meanwhile, the initiation and propagation mechanism of cracks is analyzed by means of local force chains and particle displacement vectors. However, there are few studies on the relationship between the composition of binder materials and the mechanical properties of ITZ. Therefore, establishing the relationship among composition, mesostructure, and performance will be of great significance to the improvement of the composition design principle of eco-friendly UHPC.

To propose a more scientific and effective mix design theory, this study investigated the relationship between the composition and performance of eco-friendly UHPC from a meso-mechanical point. Firstly, the DEM model of the eco-friendly UHPC matrix was constructed based on the particle flow code 3D (PFC3D). The experimental results verified the rationality of the model. On this basis, the mechanism of the effect of ITZ properties on the tensile behavior of eco-friendly UHPC matrices was studied. Then, based on the quantitative characterization of ITZ strength in the DEM model, the influence of the binder material and chemical activator on the performance of the ITZ was analyzed, and the relationship between the reactivity of the binder system and the performance of the ITZ was discussed.

## 2. Brief Description of the Experimental Test

### 2.1. Mixture Proportion and Specimen Preparation

The binder materials used in this study mainly include Ordinary Portland Cement (OPC) with a strength grade of 42.5, fly ash (FA), ground granulated blast-furnace slag (GGBS), silica fume (SF), nano aluminum oxide (NA), and nano silica (NS). The chemical composition of the material is illustrated in Table 1. SF has a density of 2.1 g/cm^3^ and a specific surface area of 20,500 m^2^/kg. GGBS has a specific surface area of 440 m^2^/kg and a density of 2.85 g/cm^3^. FA has a specific surface area of 450 m^2^/kg and a density of 2.4 g/cm^3^. The average particle size and specific surface area for NA was 20 nm and 120 m^2^/g, while for NS, it was 30 nm and 150 m^2^/g. A polycarboxylic-acid-based superplasticizer (SP) was used to obtain suitable workability. The chemical activator was analytically pure sodium sulfate (SS). Quartz sand with a different fineness (0.1–0.18, 0.18–0.48, and 0.4–0.8 mm) was used as an aggregate. The sieving results of three different grades of quartz sand are shown in Table 2. Furthermore, the mixture proportions of the eco-friendly UHPC matrix can be designed according to the method proposed in the previous study [7,32] and are listed in Table 3. The design approach of the binder system for eco-efficient UHPC is to achieve a densely compacted hardened paste by optimizing the physical packing and chemical effect of the binder system. All the drying raw materials were mixed for 180 s at a low speed. Then the SP and water were added, and the mixer was kept on low speed for 3 min. After that, the mixer was kept on high speed for 2 min. Eventually, the uniform mixture was obtained, and the specimens were shaped and vibrated. The specimens were covered with plastic sheets and cured under laboratory conditions for 24 h prior to demolding. After that, the samples were cured under standard curing conditions (T = 20 ± 2 ^◦^C, RH > 95%) for 28 days.

### 2.2. Testing Methods

**Figure 1:**
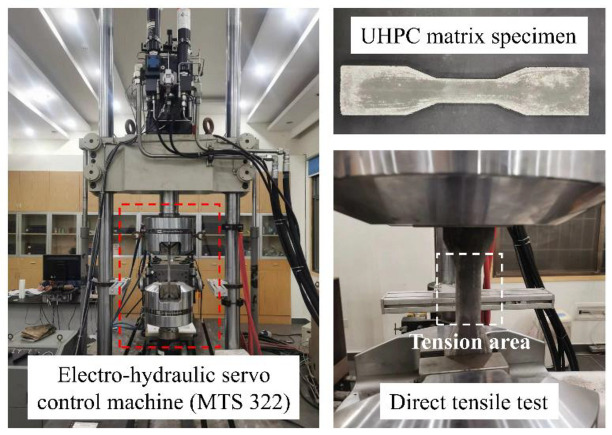
Specimen and device for testing the tensile properties of UHPC matrixes.

Compared to flexural tests, direct tensile tests can better reflect the tensile properties of the material. Therefore, this study used the direct tensile test method to investigate the tensile properties of an eco-friendly UHPC matrix. Figure 1 shows the specimen and device for testing the tensile properties. The direct tensile test was performed on an electro-hydraulic servo control machine (MTS 322) with a 450 kN load cell. The specimen was fixed by mechanical tightening of the end. The loading speed was 0.2 mm/min. The specimen was in the shape of a dog bone, with a length of 220 mm, a width of 50 mm, and a thickness of 30 mm in the tensile area. Each specimen was carefully installed and aligned by a level instrument. Two clip gauges were mounted on both sides of the specimen to obtain the tensile strain. Considering the inhomogeneity of the UHPC matrix and the representativeness of the test results, three parallel samples were tested for each mixture. The average value of two groups of similar data from three parallel samples was taken as the representative result.

## 3. Building the DEM Model of the UHPC Matrix

### 3.1. Meso-Mechanical Constitutive Model

In the bonded particle model, the complete numerical model is a composite material bonded by different kinds of particles. The macroscopic behavior of the bonded particle model is determined by the microscopic properties of the bond and particle. These bonded models exhibit different mechanical properties when transferring forces and moments between particles, and the mechanism of a contact-constitutive model will determine its applicability in concrete material simulation. Considering the feasibility and efficiency of the calculation, the DEM model of UHPC is constructed in this paper based on the three-dimensional particle flow code (PFC3D) [33]. The PFC3D uses simpler constitutive laws to represent the complex internal mechanisms of the physical model. In DEM modeling, the linear parallel bond model (LPBM) [34] is used to simulate the interactions between particles inside the concrete material.

**Figure 2:**
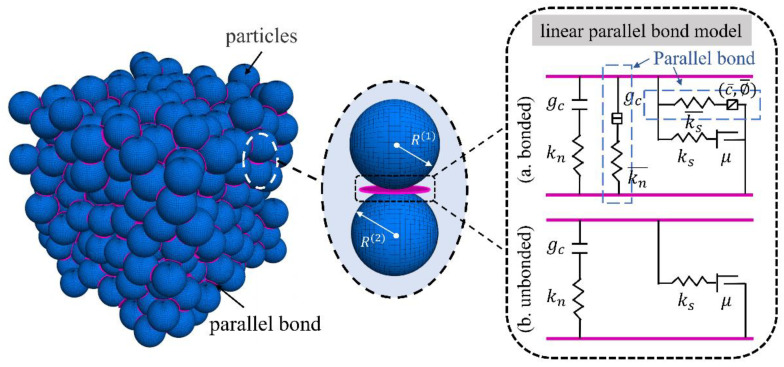
Schematic diagram of the linear parallel bond model and mechanism of action.

Figure 2 shows the action mechanism of the LPBM. In the LPBM, the contact range of the bond is a plane of finite space, which can transfer both force and moment. The force and moment of the bond can be decomposed into the normal component and shear component acting on the contact surface as follows.

(1) $$\bar{F}=-\bar{F_{n}}\hat{n_{c}}+\bar{F_{s}}$$

(2) $$\bar{M}=\bar{M_{t}}\hat{n_{c}}+\bar{M_{b}}$$

where the nst coordinate system is constructed on the basis of the contact surface, $F_{n}$ and $F_{s}$ are the normal and shear components of the force decomposition of the bond on the contact surface, respectively, and $F_{n}$ > 0 is the tensile force, and the parallel-bond moment is resolved into a twisting $M_{t}$ and bending moment $M_{b}$.

The updates of the normal force $F_{n}$, shear force $F_{s}$, twisting $M_{t}$ and bending moment $M_{b}$ of the parallel bond with the update of the timestep during the simulation are defined as follows:

(3) $$\left\{\begin{array}{c} \bar{F_{n}}=\bar{F_{n}}+\bar{k_{n}}\bar{A}\Delta \delta_{n}\\ \bar{F_{s}}=\bar{F_{s}}-\bar{k_{s}}\bar{A}\Delta \delta_{s} \end{array}\right.$$

(4) $$\left\{\begin{array}{c} \bar{M_{b}}=\bar{M_{b}}-\bar{k_{n}}\bar{I}\Delta \theta_{b}\\ \bar{M_{t}}=\bar{M_{t}}-\bar{k_{s}}\bar{J}\Delta \theta_{t} \end{array}\right.$$

where $k_{n}$ and $k_{s}$ are the normal and shear stiffness of the parallel bond, $\Delta \delta_{n}$ and $\Delta \delta_{s}$ are the relative normal and tangential displacement increments, and $\Delta \theta_{t}$ and $\Delta \theta_{b}$ are the relative twisting and bending increments, respectively. Meanwhile, the cross-sectional area of the bond $\bar{A}$, the moment of inertia $\bar{I}$ and the polar moment of inertia $\bar{J}$ of the parallel bond cross-section are defined as follows:

(5) $$\left\{\begin{array}{c} \bar{A}=\pi {\bar{R}}^{2}\\ \bar{I}=\frac{1}{4}\pi {\bar{R}}^{4}\\ \bar{J}=\frac{1}{2}\pi {\bar{R}}^{4} \end{array}\right.$$

where $\bar{R}$ is the radius of the parallel bond and is defined as the minimum value of the radius of the particles at both ends of the bond, that is, $\bar{R}=\min\left(R^{\left(1\right)},R^{\left(2\right)}\right)$.

According to the above definition of the properties of a parallel bond, the normal stress $\bar{\sigma }$ is the sum of the stress caused by the normal force and the bending moment, and the shear stress $\bar{\tau }$ is the sum of the stress caused by the shear force and the twisting. It is expressed by the following equations:

(6) $$\left\{\begin{array}{c} \bar{\sigma }=\frac{\bar{F_{n}}}{\bar{A}}+\bar{\beta }\frac{||\bar{M_{b}}||\bar{R}}{\bar{I}}\\ \bar{\tau }=\frac{||\bar{F_{s}}||}{\bar{A}}+\bar{\beta }\frac{||\bar{M_{t}}||\bar{R}}{\bar{J}} \end{array}\right.$$

where $\bar{\beta }$ is the torque contributing factor, and its value range is [0–1].

If the maximum tensile stress of the parallel bond exceeds the tensile strength $\bar{\sigma_{c}}$ of the bond or the maximum shear stress exceeds the shear strength $\bar{\tau_{c}}$, the parallel bond will fracture, and its effect on the force and moment will not be considered.

### 3.2. DEM-Based Model Construction and Monitoring

**Figure 3:**
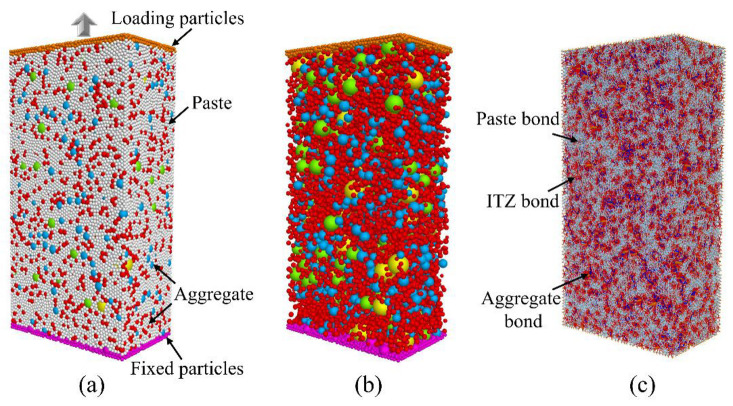
Meso-mechanics numerical model of concrete. (**a**) Meso-level components of the concrete model. (**b**) Randomly distributed aggregates. (**c**) Contact phases in DEM model.

In this paper, PFC3D is used to construct the three-phase discrete element model of an eco-friendly UHPC matrix. Among them, the paste and fine aggregates are represented by spherical particles. To realize the random distribution of particles, a rigid wall is used to establish a model boundary consistent with the size of the concrete sample. Based on the set aggregate volume fraction and gradation, all particles are randomly placed within this region. Then, “solve” program is used to set a termination condition for the concrete model building process. The “solve” logic provides a mechanism for continuously repeating the cycle model, allowing all particles in the system to move around within the boundary until a zero-stress (equilibrium) state is reached. Finally, the constructed concrete model with random distribution is shown in Figure 3a. Among them, colored particles represent aggregates, and gray particles represent cement paste. Considering that excessive particles will seriously increase the calculation time, in order to improve the calculation efficiency, the particles in the simulation are enlarged in equal proportion, which will not affect the accuracy of the simulation results under the premise of reasonably reflecting the cement paste properties. Therefore, a total of 75,282 paste particles with particle sizes of 0.5–0.83 mm and 11,839 fine aggregate particles with particle sizes of 0.9–4.75 mm are generated in the DEM model. Meanwhile, to realize the application of tensile load, the bottom particle displacement is constrained during the simulation, and the top particles are subjected to a constant axial tensile velocity to simulate the loading process.

As shown in Figure 3b, the particle size distribution of the aggregates is characterized by the sieving method. The amount of aggregate within a specific graded section is determined by experimental data. In this study, the particle size distribution is reconstructed by using the gradation curve obtained in the experiment. Different colors represent aggregate particles with different particle size ranges. In addition, considering the different bond properties of aggregate and paste, different micro-parameters are set to more realistically reflect the characteristics of internal stress and deformation of concrete in the actual loading process. As shown in Figure 3c, by identifying the types of particles at both ends of the bond, the bond types are divided into aggregate bond, paste bond, and ITZ bond. It should be noted that the contact between aggregate particles only transmits contact forces without bond properties. Therefore, a linear contact model [35] is adopted to simulate the contact behavior between aggregates. On this basis, the strain data is obtained by monitoring the displacement of the upper particle, the stress data of the model is obtained by setting a measurement circle in the center of the specimen to monitor the force of the particle within the range, and the number of microcracks is monitored by identifying the fracture of the bond.

### 3.3. Mesoscopic Parameter Calibration and Verification

Due to the heterogeneity and randomness of concrete, the results of mechanical response are obtained under the condition that all parameters cannot be kept constant. Therefore, it is difficult to obtain the influences of specific parameters independently in laboratory tests. In the DEM, it is very important to study the sensitivity of meso-parameters to the macroscopic mechanical properties of concrete before the calibration step of meso-parameters. According to the experience of mesoscopic parameter matching and previous research results [36,37], the macroscopic elastic modulus is mainly controlled by the effective modulus of the bond, the macroscopic Poisson’s ratio is mainly controlled by the stiffness ratio of the bond, and the peak strength of the material is mainly affected by the normal and tangential strength of the bond. Based on the above relationship between macroscopic and mesoscopic parameters of concrete, the following procedure can be used to calibrate the mesoscopic parameters of the concrete DEM model. The calibration procedure is as follows:

(I) Rough calibration: Firstly, the elastic modulus of concrete is roughly matched, and the effective modulus of particles and bonds in the numerical model is adjusted to obtain the elastic modulus that roughly matches that of the laboratory test of concrete. Then the Poisson’s ratio obtained from the simulation is roughly matched with that from the laboratory test by adjusting the stiffness ratio of particles and parallel bonds in the numerical model. Finally, the tensile strength and cohesion of parallel bonds are adjusted respectively to obtain the peak strength similar to the laboratory test;
(II) Accurate calibration: The accurate calibration of the mesoscopic parameters is carried out by the “trial and error method”, and the results of the PFC simulation are matched with the results of the macroscopic mechanical parameters of concrete in laboratory tests through repeated adjustment of the meso-parameters. Finally, a set of parallel bond mesoscopic parameters that well reflect the mechanical properties of concrete are obtained, as shown in Table 4.

**Figure 4:**
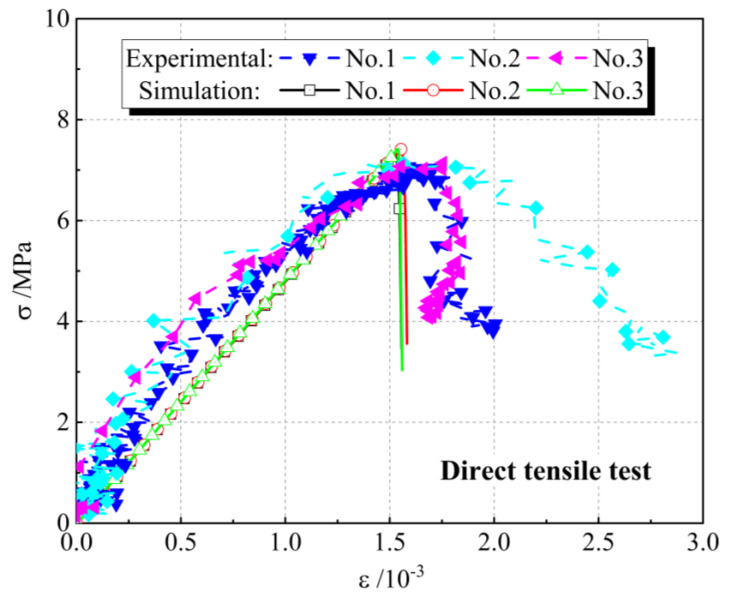
The comparison of simulation and experimental tensile stressstrain curves of UHPC matrix.

**Figure 5:**
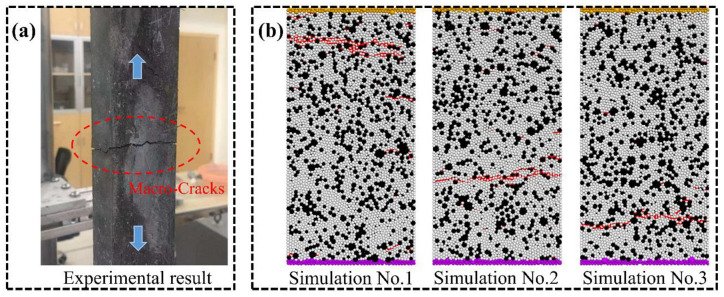
Failure patterns of samples under direct tensile loading. (**a**) Experiment. (**b**) simulations.

To validate the meso-parameters of the discrete element model, direct tensile tests are performed on the complete specimens. Meanwhile, to exclude the influence of the distribution of particles within the model on the validation results, three different seeds for the random-number generator are set up in the validation tests, and the value of the random-number seed could precisely recreate the original model; that is, three random-number seeds represent concrete samples with three different particle arrangements (Simulation No.1, No.2, and No.3). In the simulation test, the velocity of the fixed particle at the lower end of the model is set to 0, and the tension rate of the loading particles at the upper end is set to 0.001 m/s. It should be noted that the loading rates in the simulations are different from those in the laboratory experiments. In this paper, the particle displacement update is converted by a time-stepping algorithm. For example, considering the time step of 3.95 × 10^−8^ s/step in this simulation, it takes more than 250,000 steps for the loading wall to move 1 mm, which is slow enough to ensure that the specimen is in a quasi-static equilibrium state during each step of the entire tensile test. Although it can more closely meet the quasi-static requirements at a lower loading rate, it consumes a lot of calculation time. Figure 4 shows the comparison of tensile stress-strain curves obtained by laboratory experiments and numerical simulations under different random-number seeds. It can be seen that the macroscopic mechanical properties of the concrete specimens obtained from the simulations are in general agreement with the results of the laboratory experiments. Meanwhile, by comparing the simulated macro mechanical parameters with the laboratory test results, the deviation of macroscopic mechanical parameters in both the simulation and the laboratory test is less than 5%, which indicates that this set of microscopic parameters can well reflect the real mechanical properties of UHPC matrixes. In the simulations, the reproducibility of the meso-parameters in the DEM model for the mechanical properties of the UHPC matrix is verified by using the same material parameters in each set for fracture simulations of specimens with different particle distributions. To further verify the reliability of the meso-parameters, the crack propagation diagram of concrete in the direct tensile test is presented in Figure 5. It can be observed that under uniaxial tensile load, concrete samples with different random-number seeds all show the same failure pattern, which is the development of microcracks in the specimens along the aggregate edge and generates one obvious macroscopic crack, and the path of macro-cracks is exactly the same as the experiment. Meanwhile, in other areas of the specimens, only a few individual microcracks appeared in the ITZ area without forming an obvious macroscopic failure. The simulated crack expansion is consistent with the experimental results.

## 4. Results and Discussion

### 4.1. The Effect of ITZ Property on Tensile Behavior

**Figure 6:**
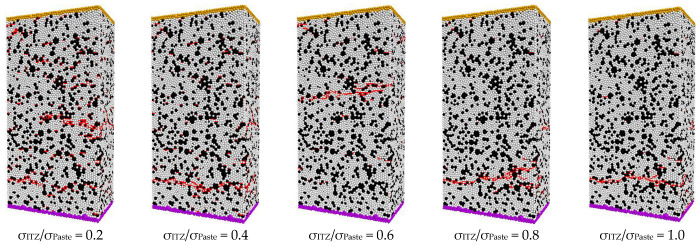
The failure patterns of UHPC matrix with different ITZ properties under direct tensile load in simulations.

Figure 6 presents failure patterns of the UHPC matrix with different ITZ properties under direct tensile loading in simulations. The simulated results indicate that the cracking behavior of the UHPC matrix is clearly influenced by the strength of ITZ. As shown in Figure 6, multiple cracks will be generated in the UHPC matrix with σ_ITZ_/σ_Paste_ = 0.2 under direct tensile loading. The distribution of cracks in the matrix is more dispersed for the UHPC with low strength of ITZ. With the improvement of the strength of ITZ, the damage pattern of the UHPC matrix gradually changed from multiple cracks to single cracks carried out.

**Figure 7:**
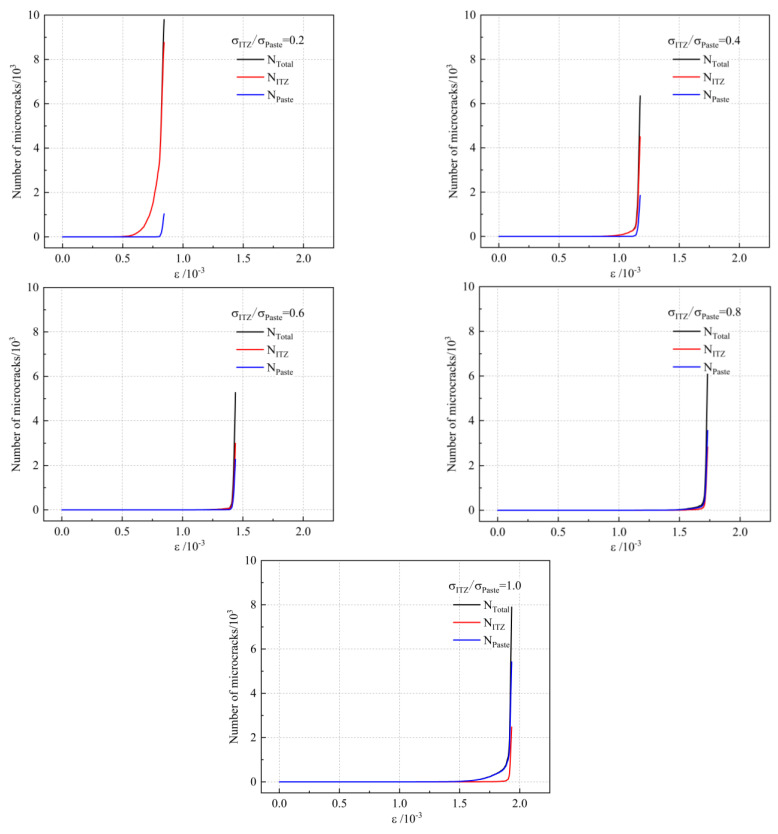
Variation curves of the number of different kinds of microcracks in UHPC matrix with ITZ strength.

The number of different kinds of microcracks in the UHPC matrix with different ITZ strengths is shown in Figure 7. For UHPC or normal concrete, microcracks generally bypass the aggregate [38]. Therefore, microcracks are mainly found in the paste and ITZ. As the strength of ITZ increases, the strain and stress required for the appearance of microcracks gradually increase. The number of microcracks will tend to decrease significantly and then increase slightly with the increase of ITZ strength. When σ_ITZ_/σ_Paste_ is between 0.6 and 0.8, the number of cracks is relatively small. This indicates that UHPC matrices with low ITZ strength are more prone to cracking and have a relatively high number of microcracks. The location where microcracks appear in the UHPC matrix changes when the ITZ strength is increased. When σ_ITZ_/σ_Paste_ is 0.2, the microcracks mainly appear in the ITZ area. This is because crack formation and development occur mainly in weak zones. As the ITZ strength increases, the percentage of microcracks appearing in the paste will gradually increase. When the strength of the ITZ is similar to the strength of the paste, the crack will not occur in a location that tends to be in a specific area, and the crack will occur mainly in the paste due to the high-volume fraction of the paste. When a UHPC matrix with low ITZ strength is cracked, the number of microcracks increases at a relatively low rate with stress. However, the cracking behavior of the UHPC matrix with high ITZ strength is different, and the number of microcracks will increase dramatically after cracking. This indicates that the brittleness of the UHPC matrix will increase as the ITZ strength increases. Compared to normal concrete, UHPC will have a significant improvement in its ITZ properties due to the use of a large number of reactive powders such as silica fume. This improvement will result in a UHPC matrix with greater brittleness. Therefore, the reason for the increase in brittleness due to increasing concrete strength is the change in ITZ properties.

**Figure 8:**
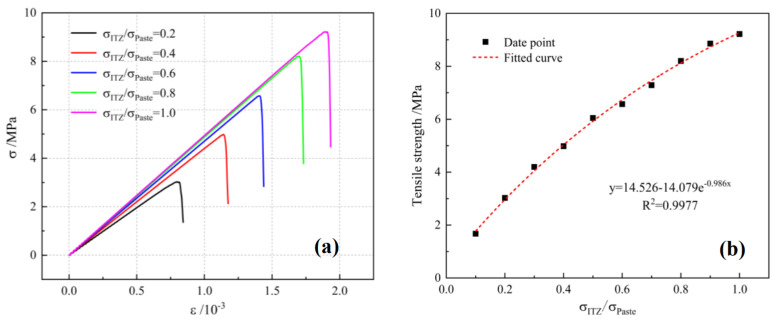
(**a**)Tensile stress–strain curves of UHPC matrix with different ITZ strengths. (**b**) Relationship between ITZ strength and tensile strength of UHPC matrix.

The stress–strain curves of UHPC with different ITZ strengths are shown and compared in Figure 8a. The properties of ITZ have a considerable effect on the tensile behavior of the UHPC matrix. The modulus of elasticity of UHPC increases as the ITZ strength increases. Figure 8b shows the relationship between ITZ strength and tensile strength of the UHPC matrix. The tensile strength of the UHPC matrix will increase with the increase of ITZ strength, but the rate of increase keeps decreasing. It is worth noting that the tensile strength of UHPC will be increased by 48% when the ITZ property is changed from normal condition to perfect. The tensile strength of normal concrete will be increased by 29% when the property of ITZ between coarse aggregate and mortar is changed from normal condition to perfect [39]. This indicates that the effect of ITZ on the tensile strength of UHPC is more significant than normal concrete. In addition, the post-peak stage of the tensile stress–strain curve becomes steeper when the ITZ strength is higher, again indicating that the increased ITZ strength leads to increased brittleness of the UHPC matrix.

### 4.2. The Effect of Binder Composition on ITZ Property and Tensile Behavior

**Figure 9:**
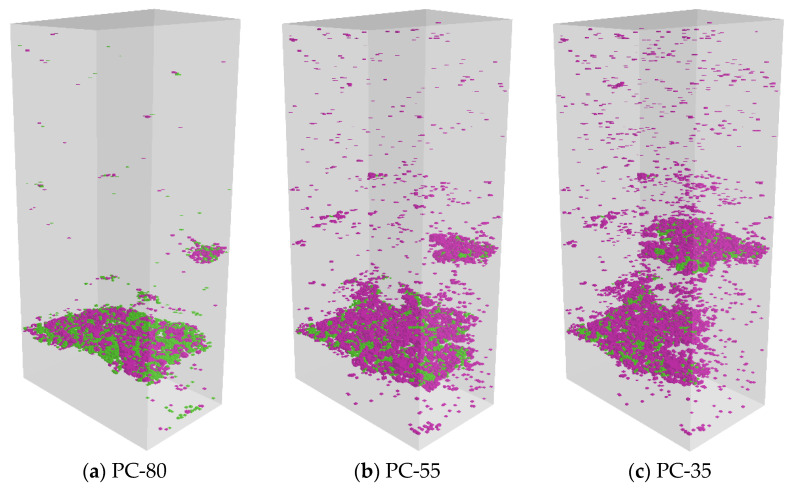
The microcrack distribution of UHPC matrix with different binder systems under direct tensile load.

The environmental impact of UHPC can be effectively reduced by the replacement of cement with mineral admixtures [32]. However, the effect of cement content on the tensile performance of UHPC is more significant than compressive performance [8]. Figure 9 presents the microcrack distribution of the UHPC matrix with different binder systems under direct tensile loading. Among them, pink indicates ITZ microcracks, and green indicates paste microcracks. It can be observed that UHPC with high cement content (PC-80) mainly generates a main crack at the time of damage, and many paste microcracks are distributed in the main fracture region. As the amount of cement decreases, the number of cracks in the UHPC matrix will tend to increase, and the failure mode of the specimen changes from a single main crack to multiple cracks. Meanwhile, the proportion of ITZ microcracks increased significantly, and the distribution area became more extensive.

The ITZ strength and tensile strength of different UHPC matrices were obtained based on experiments and numerical simulations, as shown in Table 5. From the results, it can be found that the tensile strength of the UHPC matrix will show a decreasing trend with the decrease in cement content. The porosity of the UHPC paste at 28 days under standard curing was measured by Mercury intrusion porosimetry (MIP), as shown in Table 6. Further details of MIPs testing for paste porosity can be found in previous studies [8]. Concrete is a porous material, and porosity is a key factor affecting the performance of the material. The designed UHPC paste all have very low porosity (less than 2%), which is much lower than conventional concrete paste (around 10–20%) [40,41]. The cement content was reduced from 80% to 35%, and the porosity of the paste still showed a small reduction. Therefore, the reduction in cement content did not adversely affect the performance of the paste. As shown in Table 5, the reduction of cement content in UHPC leads to a reduction of σ_ITZ_/σ_Paste_ from 0.7 to 0.32. The reduction in ITZ strength will be the main reason for the reduction of the tensile strength of the UHPC matrix.

Nanomaterials can be used for the modification of UHPC [42]. According to the results shown in Table 5, the UHPC matrix with nanomaterials has higher tensile strength than the UHPC matrix without nanomaterials. It can be found from Table 6 that the incorporation of NS and NA in UHPC with very low cement content resulted in a further reduction of the porosity of the paste. This phenomenon is mainly attributed to the nucleation effect and physical filling effect of nanomaterials. These effects lead to an increase in the initial packing density and the production of hydration products of UHPC, which in turn leads to an increase in the density of the hardened paste. It is worth noting that nanomaterials can not only change the properties of the paste, but also the properties of ITZ. It was confirmed by fiber pull-out experiments that nanomaterials incorporated into UHPC can improve the performance of ITZ between fiber and matrix [43]. The region of ITZ between paste and quartz sand has a higher porosity compared to the paste, and the ITZ structure will become denser with increasing hydration degree [38]. By the results of numerical simulations, as shown in Table 5, the strength of ITZ between paste and quartz sand in UHPC with very low cement content was improved after the incorporation of nanomaterials. Compared to NS, the improvement of ITZ performance by NA is more significant. The effects of NS and NA on the porosity of UHPC paste are similar. This suggests that the improved ITZ properties due to the incorporation of nanomaterials are responsible for the improved tensile properties of the UHPC matrix.

### 4.3. The Effect of Chemical Activator on ITZ Property and Tensile Behavior

Compared with UHPC, the binder system of eco-friendly UHPC contains a large amount of mineral admixture, which will lead to a relatively low generation of hydration products, and thus its mechanical properties are relatively low. The hydration characteristics of eco-friendly UHPC were regulated by a chemical activator to play a role in modifying the properties of eco-friendly UHPC. As shown in Table 5, the matrix of group PC-35-AA has higher tensile strength than group PC-35. This indicates that chemical activators can improve the tensile properties of eco-friendly UHPC. It is worth noting that the initial packing density will be low due to the incompatibility of the chemical activator with the SP, which in turn causes the porosity of the paste to increase, but it is still less than 2%. This indicates that although the incorporation of the chemical activator has a significant effect on the hydrate phase [8], it has no improvement in the strength of the paste. As shown in Table 5, the σ_ITZ_/σ_Paste_ is increased from 0.32 to 0.59 by incorporating the chemical activator into eco-friendly UHPC. The improvement in the tensile properties of the matrix for eco-friendly UHPC with chemical activator is mainly due to the improvement in the properties of the ITZ.

### 4.4. Discussion

ITZ is the weak link of concrete, and it has a very significant impact on the performance of concrete. UHPC reduces the amount of ITZ and improves the performance of ITZ by removing coarse aggregates, reducing the water-to-binder ratio, and using a large amount of ultra-fine reactive. The ITZ in UHPC is very dense, which is very significantly different from the ITZ in ordinary concrete [44,45]. This may make the effect of ITZ on UHPC performance less effective. However, it is clear from the content of Section 4.1 that the effect of ITZ on the tensile strength of the UHPC matrix is more significant than normal concrete. This shows that the importance of ITZ to performance does not diminish with the improvement of its own properties for high or ultra-high-strength concrete. Under direct tensile loading, the brittleness of the UHPC matrix will increase with increasing ITZ strength. From the perspective of meso-mechanical, it is revealed that the brittleness of concrete increases with strength as a consequence of the increase in ITZ strength.

As can be seen from Section 4.2, changes in the composition of the binder material system did not result in significant changes in the properties of the UHPC paste and aggregates. The change in the composition of the binder material system has resulted in a change in the hydrate phase of UHPC. This change leads to a significant alteration in the ITZ properties, which in turn affects the tensile properties of the UHPC matrix. The packing state and hydration reactions of the binder material determine the formation and development of the ITZ structure, which in turn influences the performance of the ITZ. ITZ structure in UHPC will become denser with increased hydration degree [38]. This indicates that the performance of ITZ will increase with the hydration reaction activity of the binder material system. The evaluation and calculation of the reactivity index of the binder material system are given in a previous study [7]. The reactivity indexes for PC-80, PC-55, and PC-35 were 76, 60.75, and 52.75, respectively, based on this calculation. Combined with Table 5, it can be seen that the ITZ strength of UHPC increases with increasing reactivity index. This indicates that increasing the reactivity of the binder material system can lead to an improvement in the performance of ITZ. Chemical activator enhances the reactivity of binder materials, especially mineral admixtures. The chemical activator leads to an increase in the reactivity of the binder material system for eco-friendly UHPC and, thus, to an increase in the performance of the ITZ. As a result, the tensile properties of PC-35-AA are improved compared to the control group (PC-35). This further confirms the influence of the reactivity of the binder material system on the performance of the ITZ.

Structures and buildings made of UHPC are expected to have excellent durability and a long life cycle due to the excellent mechanical properties and the excellent durability of UHPC. However, if cracking occurs in the structure, it will significantly reduce the contribution of UHPC’s excellent durability to the durability of the structure, which will defeat the original design intent. Increasing the initial cracking strength of UHPC will help to reduce the risk of cracking in the structure. Improved tensile properties of the matrix will significantly affect the initial cracking strength of UHPC [8,46]. Therefore, the relationship between the structure and properties of the UHPC matrix had been established from a meso-mechanical point through a 3D discrete element model. The relationship between the reactivity of the binder system and the properties of ITZ was analyzed. This would give guidance for the optimization of the binder system composition for eco-friendly UHPC. At the same time, the feasibility of modulating the activity of the binder material to improve ITZ properties was experimentally confirmed using nanomaterials and chemical activators as a typical method.

## 5. Conclusions

The microstructure property was quantitatively characterized using DEM with a UHPC matrix as the object of this study. The relationship between the composition of the binder system, the ITZ strength, and the tensile properties of the eco-friendly UHPC was analyzed. The main conclusions are as follows:

(1) The increase in ITZ strength will result in a decrease in the total number of microcracks. The percentage of cracks in the paste will increase as the ITZ strength increases. The tensile properties of UHPC will increase as the ITZ strength increases, but the growth rate will gradually decrease. The brittleness of UHPC will increase as the ITZ strength increases. The effect of ITZ on the tensile properties of UHPC is more significant than that of normal concrete. The tensile strength of UHPC will be increased by 48% when the ITZ property is changed from normal condition to perfect;
(2) The cement content in UHPC was reduced from 80% to 35%, and the σ_ITZ_/σ_Paste_ was reduced from 0.7 to 0.32. The ITZ strength and tensile properties of eco-friendly UHPC matrix tend to decrease as the cement content decreases, while the number of microcracks tends to increase. Nanomaterials improve the tensile properties of eco-friendly UHPC by increasing the strength of ITZ and the denseness of the paste. Compared to NS, the improvement in tensile properties of NA is more significant. The chemical activator mainly improves the tensile properties of eco-friendly UHPC by improving the ITZ properties;
(3) The hydration activity of the binder system has a significant influence on the ITZ strength, which increases with hydration activity. Both nanomaterials and chemical activators have the effect of promoting the hydration reaction of the binder material, which in turn leads to better ITZ performance of eco-friendly UHPC.

In this study, the relationship between material composition, mesostructure, and performance is analyzed by DEM, which can support the improvement of the composition design principle of eco-friendly UHPC. The disadvantage is that the simulation does not consider the real shape of the aggregate, and the accuracy of the model can be improved. Moreover, in future research, the OPC or conventional concrete will consider replacing it with its most eco-friendly option, i.e., geopolymers. This class of materials shows better performance than OPC in severe conditions.

## Figures and Tables

**Table 1:** The chemical compositions of binder materials.

Raw Materials	Content/% by Weight							Ignition Loss/%
	SiO_2_	Al_2_O_3_	Fe_x_O_y_	CaO	MgO	SO_3_	f-CaO	
OPC	22.01	4.41	3.12	62.48	2.26	2.59	0.77	2.02
FA	52.60	25.90	9.60	3.80	1.20	0.20	--	4.10
GGBS	26.15	13.70	13.90	33.50	8.00	--	--	3.50
SF	92.41	--	0.15	0.57	0.36	0.04	--	2.40
NS	99.8	--	--	--	--	--	--	0
NA	--	99.9	--	--	--	--	--	0

**Table 2:** Sieving results for three different grades of quartz sand.

Opening Size (mm)	Coarse Sand (%)	Medium Sand (%)	Fine Sand (%)
2.5	0	0	0
1.18	13.26	0	0
0.63	84.44	0	0
0.315	2.2	73.98	0
0.16	0	22.81	42.56
<0.16	0	3.21	57.44

**Table 3:** Mixture proportions of eco-friendly UHPC matrix (kg/m^3^).

	PC-80	PC-55	PC-35	PC-35-NS	PC-35-NA	PC-35-AA
OPC	856	577	364	359	359	362
GGBS	-	105	312	307	307	311
SF	214	210	208	205	205	207
FA	-	157	156	154	154	155
nano	-	-	-	15.6 (NS)	15.6 (NA)	-
SS	-	-	-	-	-	5.2
Water	182	178	177	177	177	177
SP	24.6	15.7	8.8	11.4	11.4	13.5
Sand	1177	1154	1145	1145	1145	1145

**Table 4:** Mesoscopic parameters of the simulated numerical model.

Linear Parallel Bond Model Meso-Parameters	Parameter Types	
	Paste	ITZ
Bond effective modulus $\bar{E_{c}}/GPa$	43	30
Normal-to-shear stiffness ratio $\bar{k_{n}}/\bar{k_{s}}$	1.5	1.5
Cohesion $\bar{C}/MPa$	34	23.8
Tensile strength $\bar{\sigma_{c}}/MPa$	10.7	7.5
Friction angle $tan\bar{\emptyset }/\left({}^{\circ}\right)$	30	30
Friction coefficient μ	0.5	0.5

**Table 5:** The ITZ strength and tensile strength of different UHPC matrices.

	PC-80	PC-55	PC-35	PC-35-NS	PC-35-NA	PC-35-AA
Tensile strength of the matrix (Experiment)	7.3 MPa	4.7 MPa	4.2 MPa	4.7 MPa	7.0 MPa	6.7 MPa
σ_ITZ_/σ_Paste_ (Simulation)	0.7	0.37	0.32	0.37	0.63	0.59

**Table 6:** The porosity of different UHPC pastes.

	PC-80	PC-55	PC-35	PC-35-NS	PC-35-NA	PC-35-AA
Paste porosity	1.65%	1.12%	1.50%	0.77%	0.79%	1.96%
